# The influence of maternal anemia on neonatal neurodevelopment: a systematic review and meta-analysis

**DOI:** 10.2478/abm-2026-0010

**Published:** 2026-04-30

**Authors:** Fengying He, Chiqiong Liu

**Affiliations:** Hunan Polytechnic of Environment and Biology, No. 165 Wangcheng Road, Hengyang City, Hunan 421005, China

**Keywords:** anemia, cognitive outcome, meta-analysis, neurodevelopment, pregnancy

## Abstract

**Background:**

Maternal anemia, particularly iron deficiency anemia (IDA), is a significant global health concern, affecting approximately 30% of women worldwide. Iron plays a crucial role in fetal brain development, influencing processes such as myelination, neurotransmitter synthesis, and oxygen transport. Despite theoretical links between prenatal iron deficiency (ID) and neurodevelopment, its actual effects on neonatal cognitive, motor, and socioemotional outcomes remain unclear. This study systematically evaluates the impact of maternal anemia on neonatal neurodevelopment.

**Methods:**

A systematic literature review was conducted using PubMed, Scopus, and Web of Science, covering studies published until December 2024. Keywords related to prenatal ID, maternal anemia, and neonatal neurodevelopment were used. Eligible studies focused on maternal iron levels and neonatal outcomes, excluding animal studies, non-English publications, and reviews. Out of 1,388 screened articles, 6 studies were selected for meta-analysis. Data were synthesized using standardized methods, and pooled effect sizes were calculated. Study quality was assessed using the Newcastle-Ottawa Scale.

**Results:**

The meta-analysis included 6 studies, with sample sizes ranging from 178 to 636 participants. Neonatal outcomes were assessed using the Neonatal Behavioral Assessment Scale (NBAS), Bayley Scales of Infant Development, and Mullen Scales of Early Learning. No significant correlation was found between maternal anemia and neonatal outcomes, including habituation (*P* = 0.93), orientation (*P* = 0.76), state regulation (*P* = 0.90), motor maturity (*P* = 0.71), autonomic stability (*P* = 0.10), early learning composite (*P* = 0.65), and gross motor skills (*P* = 0.59). Heterogeneity was low to moderate, and no significant publication bias was detected. Variability in iron supplementation did not yield consistent benefits.

**Conclusion:**

Maternal anemia does not significantly impact early neonatal neurodevelopment. While additional studies are unlikely to reveal meaningful effects during the neonatal period, the potential for delayed or long-term neurodevelopmental impacts remains uncertain and warrants further longitudinal research.

Iron is an essential micronutrient for human growth and development, playing a critical role in physiological processes, including oxygen transport, cellular metabolism, myelination, and neurotransmitter synthesis [1]. During pregnancy, the demand for iron increases significantly to support the developing fetus and the physiological changes in the mother [2, 3]. The prenatal period is a critical phase for fetal brain development, with a high rate of growth and differentiation occurring during it. Iron deficiency (ID) during this period can have profound effects on fetal neurodevelopment, potentially leading to long-term deficits [4]. Despite being preventable, ID and iron deficiency anemia (IDA) remain among the most prevalent nutritional deficiencies worldwide, particularly in low- and middle-income countries.

The prevalence of anemia is disproportionately higher in low-income regions, where malnutrition and infectious diseases are prevalent. However, even in high-income countries, risk factors such as restrictive diets, multiple pregnancies, and genetic disorders affecting iron metabolism contribute to the burden of ID [5, 6].

Each trimester of pregnancy plays a distinct and critical role in the development of the central nervous system (CNS). In the first trimester, the foundation of the CNS is laid as neural tube formation, and the initial organization of brain structures occurs. This stage sets the ground for all the following brain development. During the second trimester, the brain enters a phase of rapid growth, marked by the proliferation of neurons and the beginning of synaptogenesis, where neural connections start to form. By the third trimester, the focus shifts to the maturation of neural circuits, synaptic development, and the onset of myelination [7, 8]. Myelination is critical for efficient signal transmission within the brain and is heavily dependent on iron. Iron, as a cofactor for enzymes involved in neurotransmitter synthesis, such as dopamine and serotonin, as well as mitochondrial energy production, is necessary throughout these stages to support the complex processes driving brain growth [5, 9, 10].

During pregnancy, the fetus depends entirely on maternal iron stores to meet its developmental needs. Insufficient maternal iron levels can lead to reduced oxygen delivery due to low hemoglobin, impairing fetal brain development. Moreover, ID can directly affect the biochemical pathways critical for neuronal growth and synaptic plasticity [11, 12]. The relationship between prenatal ID and impaired neurodevelopment in neonates is multifactorial. One primary mechanism through hypoxia: Chronic hypoxia can have detrimental effects on brain development, particularly in regions such as the prefrontal cortex, which is highly sensitive to oxygen deprivation. It is important to note that genetic and socioeconomic factors play an important role in neurodevelopment, which cannot be foreseen when trying to understand human neurodevelopment [2, 13, 14].

In addition to hypoxia, ID disrupts several critical biological processes within the CNS. Neurotransmitter synthesis, essential for regulating mood, cognition, and behavior, is compromised by insufficient iron. Dopamine, serotonin, and gamma-aminobutyric acid (GABA), all dependent on iron for their production, are required for cognitive and emotional functioning. Furthermore, ID impairs the function of oligodendrocytes, the cells responsible for myelination, leading to delays in the maturation of white matter tracts [15-18].

Research suggests that ID during pregnancy may also affect gene expression through epigenetic mechanisms. Alterations in DNA methylation and histone modification caused by low iron levels may disrupt the expression of genes necessary for brain development [19-21].

The prevalence of ID is particularly concerning in populations already facing socioeconomic disadvantages; pregnant adolescents, women with high parity, and those with inadequate access to healthcare are at increased risk. Since the association between prenatal ID and neurodevelopmental outcomes is not well known, further research is needed to explain the precise mechanisms involved and identify the extent to which it can affect the neurodevelopment of the offspring.

This study aims to examine whether there is any association between prenatal anemia and, if there is, the extent to which prenatal anemia can affect neonatal neurodevelopment.

## Methods

### Research question and search strategy

The meta-analysis aimed to address the question: “How does prenatal ID and anemia affect neonatal neurodevelopmental outcomes?” A systematic search of various electronic databases, such as PubMed, Scopus, and Web of Science, was performed to locate pertinent studies published until December 2024. The search utilized keywords and medical subject headings (MeSH) like “prenatal iron deficiency,” “maternal anemia,” “neurodevelopment,” “cognitive outcomes,” and “neonatal outcomes.” Boolean operators were used for the effective combination of terms. Additionally, the reference lists from the selected articles were manually examined for other relevant studies.

### Inclusion and exclusion criteria

Studies were included based on the following criteria: they needed to examine the link between prenatal ID or anemia and neonatal neurodevelopmental outcomes, involve human participants, present quantitative measures of neurodevelopmental outcomes, and be published in peer-reviewed journals. Studies were excluded if they did not report neurodevelopmental outcomes, were conducted on animals, were published in a non-English language, and were review articles, case reports, or conference abstracts.

### Screening

The first step in the screening process was to eliminate duplicate records. Two researchers independently examined titles and abstracts for relevance. Full-text articles were obtained for studies that met the preliminary screening criteria and were evaluated for inclusion based on these criteria. Any discrepancies were addressed through discussion or by consulting a third-party reviewer.

### Quality assessment

The Newcastle Ottawa critical appraisal tool was employed to evaluate the quality of observational studies. This assessment scale features 8 items, categorized into 3 sections: selection of study groups, comparability of groups, and outcome ascertainment. Each item receives a maximum score of 1 point, except for comparability, which can earn up to 2 points. The cumulative score varies from 0 to 10, with higher scores indicating superior quality. Two authors conducted the quality assessment, and any conflicts were resolved through discussion.

### Data extraction and synthesis

Data was gathered utilizing a standardized data collection form. Essential details encompassed study design, sample size, characteristics of participants, maternal iron status assessments, timing of evaluations during pregnancy, neonatal neurodevelopmental outcomes, and statistical findings. The data synthesis process included summarizing results and arranging them based on outcome measures and study characteristics.

### Statistical analysis

Meta-analytic methods were utilized with both fixed and random effects models to calculate pooled effect sizes for neurodevelopmental outcomes from the included studies. The *I*^2^ statistic was used to assess heterogeneity, where values exceeding 50% suggest considerable heterogeneity. Publication bias was assessed through visual examination of funnel plots, complemented by Egger’s test. Statistical significance was determined at *P* < 0.05. All analyses were performed using the R programming language with the “meta” package 4.0.1).

## Results

### Literature screening process and results

**Figure 1.**
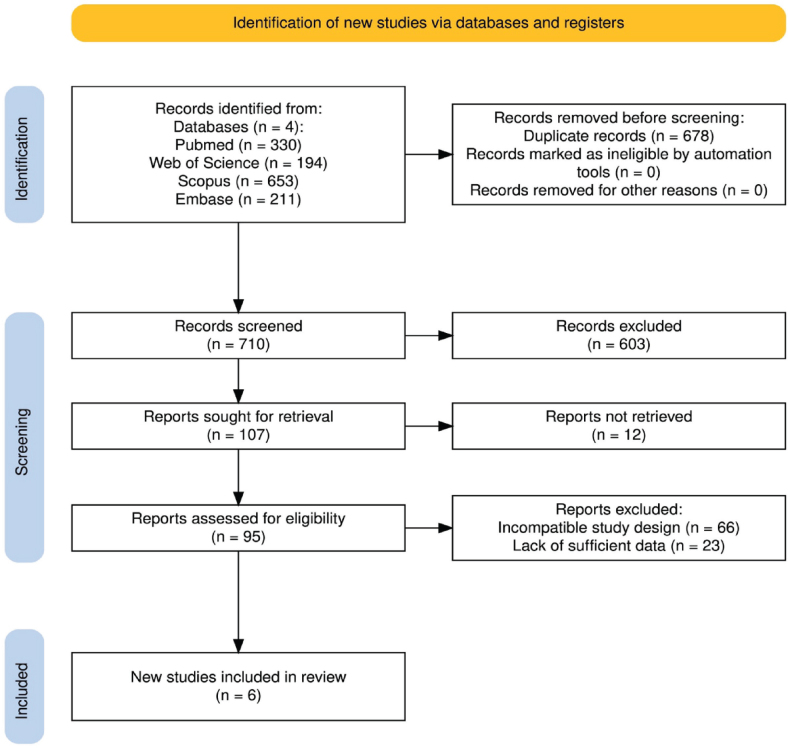
PRISMA flow diagram for the study selection process for this meta-analysis.

The initial search produced a total of 1,388 articles. After eliminating duplicates, 710 articles were left, and upon reviewing titles and abstracts for relevance, 107 were retained. Subsequently, 2 researchers performed a thorough full-text evaluation according to the established inclusion and exclusion criteria, ultimately identifying 6 articles for analysis. The process of screening is represented in the PRISMA flowchart (**Figure 1**).

### Study characteristics

The studies included in the review showed differences in location, design, and participant characteristics. Most of the research took place in Spain and Benin, utilizing prospective longitudinal and cohort study designs. Sample sizes ranged from 178 to 636 participants, with a majority consisting of singleton pregnancies. The proportion of male infants across the studies varied from 47.6% to 51.4%. Maternal iron status was evaluated through various biomarkers, such as serum ferritin, transferrin saturation, and hemoglobin levels, which were measured at distinct stages of pregnancy (first, second, and third trimesters) and at the time of delivery. Neonatal neurodevelopmental outcomes were examined using the Neonatal Behavioral Assessment Scale (NBAS), Bayley III Scales of Neurodevelopment, and the Mullen Scales of Early Learning, which were administered between 48 h after birth and up to 1 year of age. The iron supplementation protocols differed among the studies, with daily doses ranging from 43.9 mg to 48.9 mg, typically starting around the 15th week of pregnancy. Major findings indicated that there was no consistent link between maternal anemia and neonatal neurodevelopmental outcomes, although some studies noted weak correlations with specific NBAS domains that varied depending on the trimester when the assessment took place. **Table 1** provides a summary of the study characteristics.

**Table 1.** Characteristics of the included papers

Study characteristic	Location	Study design	Participants (mothers)	Fetus number	% Boys	Maternal iron assessment	Neonatal/infant assessment	Iron supplementation	Key findings
Hernández-Martínez et al. [7]	Spain	Prospective longitudinal	216	Singleton	49.5%	SF andTS at 10-15, 24-27, and 33-34 weeks of gestation	NBAS at 48-72 h postpartum	Systematic iron supplementation from -week 15, mean dose 48.9 mg/d	ID in first/second trimesters weakly predicted NBAS ANS scores; in third trimester, predicted NBAS motor, state organization, and robustness/endurance scores.
Aranda et al. [22]	Spain	Prospective longitudinal	210	Singleton	47.6%	Hb levels at third trimester and delivery	NBAS at 48-72 h postpartum	Systematic iron supplementation from -week 15, mean dose 43.9 mg/d	Hemoconcentration risk in third trimester related to decreased neonatal state regulation and alertness; at delivery, to decreased state regulation and poor robustness/endurance.
Berglund et al. [23]	Spain	Prospective observational	331	Singleton	51.4%	Ferritin, Hb, transferrin saturation at 34 weeks and at delivery	Bayley III scales of neurodevelopment at 18 months	Iron supplementation at entry (34.4% of mothers)	Maternal ID at 34 weeks associated with lower composite motor scores at 18 months; ID at delivery associated with lower cognitive, receptive, expressive, and composite language scores.
Mireku et al. [11]	Benin, Africa	Prospective cohort	636	Singleton	N/A	Hb concentration at first and second ANC visits and at delivery	Mullen scales of early learning at 1 year	Oral iron and folic acid after first ANC visit	Inverted U-shaped relationship between maternal Hband infant GM function; Hb concentration between 90 g/L and 110 g/L appears optimal for early GM function; prenatal anemia associated with higher GM scores.
Menon etal. [15]	India	Cohort study	211 (second trimester), 178 (third trimester)	Singleton	N/A	Hb, SF, sTfR at second and third trimester	Infant anthropometric data and neurobe-hav¡oral data at ~3 weeks postpartum	N/A	Infants of non-anemic mothers in the second trimester were heavier, taller and had larger head circumference. Infants of non-anemic mothers in third trimester had higher orientation scores.
Mireku et al. [24]	Benin, Africa	Prospective cohort	636	Singleton	N/A	Maternal ID was assessed through blood samples taken during the prenatal period	CBSF concentration, Mullen Scales of early learning at 1 year	Oral iron and folic acid after first ANC visit	No association between prenatal ID and CBSF levels or infant cognitive and motor development at 1 year of age. However, possession score and maternal education were related to cognitive development in the infants.

1 ANS, autonomous nervous system; CBSF, cord blood serum ferritin; GM, gross motor; Hb, Hemoglobin; ID, iron deficiency; NBAS, neonatal behavior assessment scale; SF, serum ferritin; sTfR, soluble transferrin receptor;TS, transferrin saturation.

### Quality assessment

The studies included in the review were evaluated using the Newcastle-Ottawa Scale (NOS) for cohort studies. Most of them obtained the top score of 9 stars, signifying high methodological quality. Five studies satisfied all criteria, whereas Menon et al. [15] achieved 8 stars because they failed to meet 1 comparability criterion. Collectively, these studies demonstrated a low risk of bias, reinforcing the reliability of the meta-analysis findings. The summary quality assessment can be found in **Table 2**.

**Table 2.** Quality assessment table

Study	1	2	3	4	5	6	7	8	9	Total stars
Hernández-Martínez et al. [7]	Yes	Yes	Yes	Yes	Yes	Yes	Yes	Yes	Yes	9
Aranda et al. [22]	Yes	Yes	Yes	Yes	Yes	Yes	Yes	Yes	Yes	9
Berglund et al. [23]	Yes	Yes	Yes	Yes	Yes	Yes	Yes	Yes	Yes	9
Mireku et al. [11]	Yes	Yes	Yes	Yes	Yes	Yes	Yes	Yes	Yes	9
Menon et al. [15]	Yes	Yes	Yes	Yes	Yes	-	Yes	Yes	Yes	8
Mireku et al. [24]	Yes	Yes	Yes	Yes	Yes	Yes	Yes	Yes	Yes	9

1 1) Representativeness of exposed cohort; 2) Selection of non-exposed cohort; 3) Ascertainment of exposure; 4) Outcome not present at start; 5) Comparability – most important factor; 6) Comparability – additional factor; 7) Assessment of outcome; 8) Adequate follow-up; 9) Adequacy of follow-up.

### Neonatal behavioral assessment scale (NBAS)

#### Habituation

**Figure 2.**
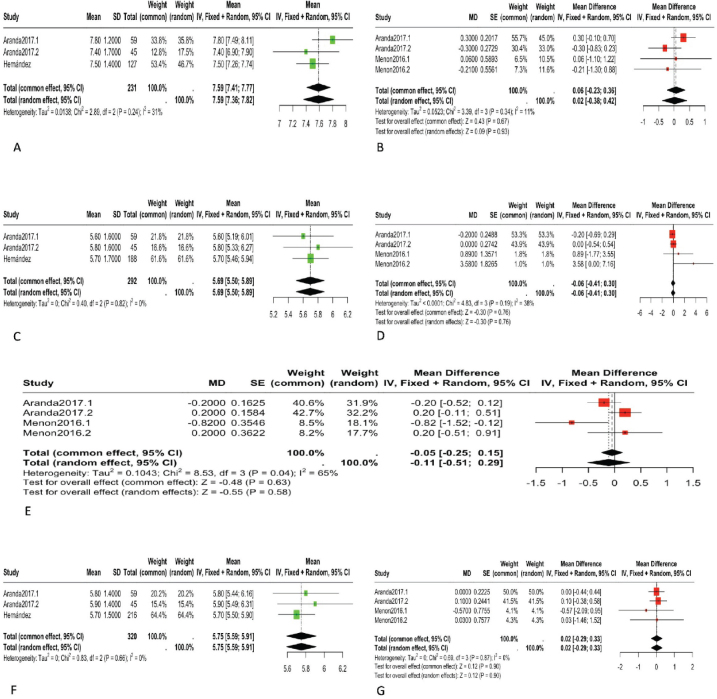
Results of the NBAS meta-analysis **(A)** forest plot displaying NBAS habituation scores across the studied samples. **(B)** A comparison of habituation scores between neonates from mothers with or without anemia. **(C)** Forest plot illustrating NBAS orientation scores. **(D)** A comparison of orientation scores between neonates from anemic and non-anemic mothers. **(E)** Forest plot showing the range of state scores from the NBAS. **(F)** Forest plot depicting the regulation of state scores in the NBAS. **(G)** A comparison of the regulation of state scores between neonates from anemic and non-anemic mothers. CI, confidence interval; NBAS, Neonatal Behavioral Assessment Scale; SD, standard deviation; SE, standard error.

The meta-analysis assessing NBAS habituation scores involved 3 studies encompassing a total of 231 observations. The common effect model produced a mean score of 7.59 (95% confidence interval [CI]: 7.41-7.77), while the random effects model yielded a nearly identical mean score of 7.59 (95% CI: 7.36-7.82; see **Figure 2A**). There was moderate heterogeneity present (*I*^2^ = 30.9%, *P* = 0.2353), and no significant publication bias was detected (*P* = 0.92). Additionally, no outliers were identified in the analysis.

In a comparison between neonates from anemic and non-anemic mothers across 4 studies, there was no significant difference in habituation scores (MD = 0.02, 95% CI: -0.38-0.42, *P* = 0.9301, see **Figure 2B**), and low heterogeneity was observed (*I*^2^ = 11.5%, *P* = 0.3353). The analysis for publication bias using Egger’s test did not show significance (*P* = 0.56), and again, no outliers were found.

#### Orientation

For the NBAS orientation, 3 studies comprising 292 observations were examined. Both the common and random effects models yielded an average score of 5.69 (95% CI: 5.50-5.89, **Figure 2C**). The level of heterogeneity was minimal (*P* = 0.0%, *P* = 0.8173). There was no significant evidence of publication bias (*P* = 0.94). When comparing neonates of anemic and non-anemic mothers across 4 studies, the results indicated no significant difference in orientation scores (MD = –0.06, 95% CI: –0.41-0.30, *P* = 0.7609, **Figure 2D**), and moderate heterogeneity was observed (*I*^2^ = 37.8%, *P* = 0.1850). Egger’s test for publication bias also showed no significance (*P* = 0.1). Furthermore, no outliers were identified in both single-arm and double-arm analyses.

#### Range of state

The analysis of the NBAS range of states included 4 studies. The common effects model demonstrated no significant difference between neonates born to anemic versus non-anemic mothers (MD = –0.05, 95% CI: -0.25-0.15, *P* = 0.6336; see **Figure 2E**). Similarly, the random effects model indicated no significant difference (MD = -0.11, 95% CI: -0.51-0.29, *P* = 0.5814). However, there was substantial heterogeneity among the studies (*P* = 64.8%, *P* = 0.0362). The publication bias of the studies was not significant (*P* = 0.60), and no outliers were detected.

#### Regulation of state

Three studies comprising 320 observations were included in the analysis of NBAS regulation of the state. Both the common and random effects models yielded a mean score of 5.75 (95% CI: 5.59-5.91, see **Figure 2F**), with no evidence of heterogeneity (*P* = 0.0%, *P* = 0.6598). The analysis also indicated no significant publication bias among the studies (*P* = 0.15).

When comparing neonates from anemic and non-anemic mothers across 4 studies, no significant difference was found (MD = 0.02, 95% CI: –0.29-0.33, *P* = 0.9020, see **Figure 2G**), again showing no heterogeneity (*1*^2^ = 0.0%, *P* = 0.8745). The Egger’s test for publication bias was not significant (*P* = 0.36). Additionally, no outliers were detected in either the single-arm or double-arm analyses.

#### Motor maturity

**Figure 3.**
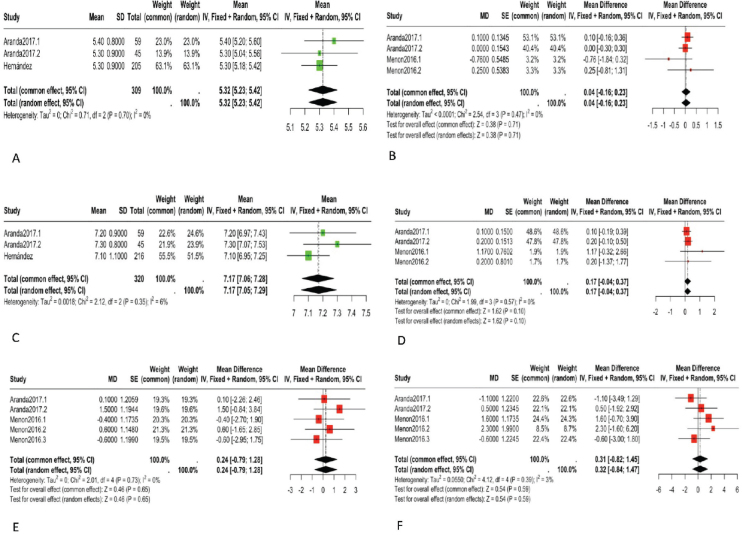
Additional meta-analysis results for neurodevelopmental outcomes. **(A)** Forest plot for NBAS motor maturity scores obtained from the studies. **(B)** A comparison of motor maturity scores between neonates from mothers with or without anemia. **(C)** Forest plot representing NBAS autonomic stability scores. **(D)** A comparison of autonomic stability scores between neonates with anemic and non-anemic mothers. **(E)** Comparative analysis of ELC scores between anemic and non-anemic neonates. **(F)** A comparison of GM scores between neonates from anemic and non-anemic mothers. CI, confidence interval; ELC, early learning composite; GM, gross motor; MD, NBAS, Neonatal Behavioral Assessment Scale; SD, standard deviation; SE, standard error.

The meta-analysis examining motor maturity in the NBAS included 3 studies with a total of 309 observations. Both the common effects and random effects models indicated a mean score of 5.32 (95% CI: 5.23-5.42, see **Figure 3A**), showing no heterogeneity (*I*^2^ = 0.0%, *P* = 0.7012). The evaluation for publication bias using Egger’s method was not significant (*P* = 0.71). When comparing neonates from anemic and non-anemic mothers across 4 studies, no significant difference was found (MD = 0.04, 95% CI: –0.16-0.23, *P* = 0.7050, see **Figure 3B**), again showing no heterogeneity (*P* = 0.0%, *P* = 0.4673). Additionally, the analysis for publication bias was not significant (*P* = 0.49), and no outliers were identified in the motor maturity assessments.

#### Autonomic stability

The analysis of NBAS autonomic stability included 3 studies with 320 observations. The common effect model produced a mean score of 7.17 (95% CI: 7.06-7.28), while the random effects model also indicated a mean score of 7.17 (95% CI: 7.05-7.29, **Figure 3C**). Heterogeneity was low, with an *P* of 5.9% (*P* = 0.3457). No publication bias was found (*P* = 0.25). A comparison between neonates from anemic and non-anemic mothers across 4 studies revealed no significant difference (MD = 0.17, 95% CI: –0.04-0.37, *P* = 0.1046, **Figure 3D**), and there was no evidence of heterogeneity (*I*^2^ = 0.0%, *P* = 0.5747). The Egger’s test for publication bias was not significant (*P* = 0.31). Additionally, no outliers were found in the autonomic stability data meta-analyses.

#### Early learning composite

The analysis of early learning composite (ELC) scores for neonates from anemic versus non-anemic mothers across 5 studies revealed no significant difference (MD = 0.24, 95% CI: -0.79-1.28, *P* = 0.6461, **Figure 3E**), and there was no heterogeneity (*I* = 0.0%, *P* = 0.7334). Additionally, no publication bias was detected among the studies (*P* = 0.66). There were no outliers identified.

#### Gross motor

The examination of gross motor (GM) scores in 5 studies showed no significant difference between neonates with anemic and non-anemic mothers (MD = 0.32, 95% CI: -0.82-1.45, *P* = 0.5891, **Figure 3F**), indicating low heterogeneity (*I*^2^ = 2.9%, *P* = 0.3900). Additionally, Egger’s test for publication bias yielded a non-significant result (*P* = 0.45), and no outliers were identified.

## Discussion

The findings of this meta-analysis suggest no significant association between prenatal ID anemia and neonatal neurodevelopmental outcomes across various standardized measures, including the Neonatal Behavioral Assessment Scale (NBAS), ELC, and GM scores. Consistently, no significant differences were observed between neonates born to anemic and non-anemic mothers across key domains such as habituation, orientation, regulation of state, motor maturity, and autonomic stability. Heterogeneity was generally low to moderate across the outcomes, and no significant publication bias was detected, further supporting the robustness of the results. These findings indicate that maternal anemia during pregnancy may have a limited impact on early neonatal neurodevelopment; however, the long-term implications and subtle neurodevelopmental effects may require further investigation through longer follow-up studies.

ID might affect neonatal neurobehavioral development, varying based on which trimester it occurs in [7, 15]. Hernández-Martínez et al. [7] reported that in the first and second trimesters, maternal ID might be related to alterations in the autonomic response, which might indicate possible detriments on brain growth. This might manifest as an increase in trembling, restlessness, and alterations in skin blood flow and, thus, skin color [7, 8, 25]. ID happening in the third trimester was observed to affect motor skills and inhibition skills [7]. Various neurodevelopmental deficits have also been noted to be altered more by maternal ID during various trimesters of pregnancy. According to Hernandez, ID occurring during the first and second trimesters has been linked to alterations in the neonate’s general autonomic response. This reflects the critical role of maternal iron levels in supporting the development of systems that regulate involuntary physiological functions [16, 26-29]. In contrast, ID during the third trimester predominantly affects motor skills and self-regulation in the neonate. Hernandez notes that third-trimester ID can impair motor performance, reducing robustness and endurance in newborns. On top of that, it has a notable impact on state organization, which is the ability of a child to regulate their physiological systems in response to external stimuli. This insists on the importance of maternal iron sufficiency throughout pregnancy to ensure optimal neurodevelopmental and physiological outcomes for the neonate [7, 30, 31].

Since neonatal behavior can be seen as a predictor of childhood temperament, difficulties in motor skills during infancy may be indicative of a difficult temperament by 4 months of age, an increased likelihood of behavioral issues during childhood, and a heightened risk of externalizing problems by the age of 6. Similarly, challenges in state organizations are associated with an elevated risk of behavioral problems and the development of attention-deficit hyperactivity disorder (ADHD) [7]. Various factors are associated with neonatal development, like socioeconomic status, demographic status, and genetic variations, which must be taken into account [11, 24].

The mechanisms by which ID can impair neonatal neurodevelopment vary in each trimester; in the first trimester, since synaptogenesis happens, ID can impair myelinization, neurotransmitter synthesis, and be detrimental to hippocampus formation [2, 4].

Animal studies have shown that fear responses in offspring born to iron-deficient mothers are altered. Offspring of iron-deficient mothers exhibit decreased fear responses, as evidenced by reduced reactions to potentially threatening stimuli. Along with reduced fear responses, these offspring also demonstrate lower levels of behavioral inhibition, which could lead to increased risk-taking behaviors with potentially harmful long-term effects. Research indicates that ID may disrupt the dopaminergic system, which is crucial for mood regulation, behavior, and cognition [15, 22, 23]. ID is also linked to reduced overall activity levels in these offspring. This decrease in activity can affect their physical development and limit exploratory behaviors, which are essential for learning about their environment [2, 10, 32]. Additionally, maternal ID significantly affects the cognitive development of offspring [9].

On the contrary, high hemoglobin levels, particularly in the third trimester of pregnancy, can have significant negative effects on neonatal neurodevelopment. Some studies have shown that high hemoglobin concentrations during the third trimester are associated with decreased state regulation and reduced alertness in newborns. Furthermore, at the time of delivery, these elevated levels are related to lower state regulation and poor scores in measures of robustness and endurance [22, 33]. Hemoconcentration occurs when there is an increase in blood viscosity, which can impair placentalfetal blood flow. As a result, the fetus may receive a reduced supply of essential oxygen and nutrients, which are crucial for normal development. In addition, high iron levels, which contribute to hemoconcentration, can lead to oxidative stress, damaging cells and tissues. These factors can severely affect the development of the fetus, particularly in terms of neurodevelopment and overall health [22, 34-36].

The relationship between maternal anemia and neonatal neurodevelopment remains complex and not fully understood, with studies showing varying results. Some studies report a direct link between maternal anemia and adverse neurodevelopmental outcomes, while others suggest an inverse or even changing relationship, indicating that high and low prenatal serum iron levels may have negative effects on neonatal brain growth [2, 36, 37]. This inconsistency highlights the intricate nature of neurodevelopment and implies that factors beyond maternal iron levels could significantly influence outcomes. There is a noticeable gap in the literature regarding the longterm effects of maternal iron status on neurodevelopment beyond early childhood. Although some studies have explored the impact during adolescence, there is limited evidence regarding outcomes in adulthood. This emphasizes the need for ongoing research to track neurodevelopmental outcomes over time. Additionally, there is insufficient evidence regarding the benefits of iron supplementation during different stages of pregnancy or at delivery in improving offspring neurodevelopment. This suggests a need for further exploration into optimal supplementation strategies, particularly to determine if specific populations would benefit from targeted interventions. It is noteworthy to note that the neurodevelopmental state of neonates depends on many variables apart from prenatal hemoglobin and iron levels, such as socioeconomic, genetic, and even the emotional state of the mother [12, 38, 39].

Our study aimed to explore the effect of prenatal ID anemia on early neonatal neurodevelopment later in life; however, the results are limited due to the scarcity of studies that objectively assess neurological function in human neonates. Given the consistently null findings in early neurodevelopmental outcomes, further studies focusing solely on the neonatal period are unlikely to yield significant results. To advance understanding in this area, future research should prioritize investigating the potential long-term effects of maternal anemia on cognitive, behavioral, and emotional development throughout childhood and beyond.

## Conclusion

The single-arm analyses yielded average scores for different NBAS domains, showing consistent results across studies with low to moderate variability. When comparing newborns from anemic and non-anemic mothers, no notable differences were observed in any of the NBAS domains, ELC, or GM scores. These results indicate that antenatal anemia may not have a significant effect on neonatal neurodevelopment outcomes as assessed by the NBAS and early learning evaluations. Although our study found no significant link between prenatal anemia and neonatal neurodevelopment, and further research limited to the neonatal period is unlikely to provide new insights, future studies should focus on long-term followup to assess potential delayed effects on child development.
